# Minimally Invasive Partial versus Radical Nephrectomy for Non-metastatic pT3a Renal Cell Carcinoma: a Multicenter Matched Cohort Study

**DOI:** 10.1590/S1677-5538.IBJU.2025.0500

**Published:** 2026-01-26

**Authors:** Xiangpeng Zou, Zhenhua Liu, Yunhan Luo, Peimin Zhou, Longbin Xiong, Zhoujie Sun, Xuesong Li, Peng Hong, Kangbo Huang, Chunsen Yang, Zhaohui Zhou, Yulu Peng, Xin Luo, Junhang Luo, Xin Yao, Shengjie Guo, Pei Dong, Hui Han, Fangjian Zhou, Shudong Zhang, Wei Yu, Zhiling Zhang

**Affiliations:** 1 Sun Yat-sen University Cancer Center Department of Urology Guangzhou P. R. China Department of Urology, Sun Yat-sen University Cancer Center, Guangzhou, P. R. China; 2 State Key Laboratory of Oncology in Southern China Collaborative Innovation Center for Cancer Medicine State Key Laboratory of Oncology in Southern China Guangzhou P. R. China State Key Laboratory of Oncology in Southern China; Collaborative Innovation Center for Cancer Medicine, Guangzhou, China; State Key Laboratory of Oncology in Southern China, Guangzhou, P. R. China; 3 Peking University First Hospital Department of Urology Beijing China Department of Urology, Peking University First Hospital, Beijing, China; 4 Peking University Institute of Urology Beijing China Institute of Urology, Peking University, Beijing, China; 5 The National Urological Cancer Center of China Beijing China The National Urological Cancer Center of China, Beijing, China; 6 Peking University Third Hospital Department of Urology Peking China Department of Urology, Peking University Third Hospital, Peking, China; 7 Tianjin Medical University Cancer Institute & Hospital Department of Urologic Oncology Tianjin China Department of Urologic Oncology, Tianjin Medical University Cancer Institute & Hospital, National Clinical Research Center for Cancer, Key Laboratory of Cancer Prevention and Therapy, Tianjin's Clinical Research Center for Cancer, Tianjin, China; 8 Sun Yat-sen University First Affiliated Hospital Department of Urology Guangzhou China Department of Urology, First Affiliated Hospital, Sun Yat-sen University, Guangzhou, China

**Keywords:** Carcinoma, Renal Cell, Nephrectomy, Minimally Invasive Surgical Procedures

## Abstract

**Purpose:**

To evaluate oncological and functional outcomes of minimally invasive partial and radical nephrectomy (MIS-PN vs. MIS-RN) in patients with pT3aN0M0 renal cell carcinoma (RCC).

**Materials and Methods:**

We performed a multicenter retrospective study of patients with pT3aN0M0 RCC treated with MIS-PN or MIS-RN. The primary outcome was recurrence-free survival (RFS). Secondary outcomes included de novo eGFR <60 mL/min/ 1.73 m^2^ (CKD-S) and <45 mL/min/ 1.73 m^2^ (CKD-S3b) at the new baseline (1-12 month postoperatively), as well as CKD-S at the latest follow-up (>1 year postoperatively). A 1:2 ratio propensity score matching (PSM) was applied to balance covariates, and inverse probability weighting (IPW) served as sensitivity analysis. Survival curves were estimated using the Kaplan-Meier method, and multivariable analyses (MVA) were performed to identify predictors of oncological and functional outcomes.

**Results:**

A total of 303 patients were enrolled (113 MIS-PN/190 MIS-RN) with a median follow-up of 39.0 months (IQR 26.8-52.9). After PSM (66 MIS-PN/54 MIS-RN), no significant difference in RFS was observed between two groups (p=0.23). MVA revealed that surgical approach was not an independent predictor of RFS (HR: 1.00, p=1.00). Among patients with available new baseline eGFR after PSM (41 MIS-PN/37 MIS-RN), MIS-RN was independently associated with a higher risk of CKD-S (OR: 7.96, p=0.03). Among patients with available the latest follow-up eGFR after PSM (41 MIS-PN/37 MIS-RN), MIS-RN remained an independent predictor of CKD-S at the latest follow-up (OR: 7.98, p=0.03). IPW analysis yielded consistent results. Additionally, IPW identified MIS-RN as an independent risk factor for CKD-S3b at the new baseline (OR: 18.29, p<0.01).

**Conclusion:**

MIS-PN provided comparable mild term oncologic outcomes to MIS-RN while offering superior renal function preservation. MIS-PN may be a viable option for selected T3a RCC patients when nephron preservation is indicated.

## INTRODUCTION

Partial nephrectomy (PN) is recommended as the preferred treatment for localized renal cell carcinoma (RCC), as it offers similar tumor control to radical nephrectomy (RN), while preserving more renal parenchyma and function(1, 2). This nephron-sparing effect has been associated with reduced cardiovascular morbidity and improved overall survival (OS) (3, 4). According to the 8th AJCC/TNM criteria (5), T3a RCC is defined as tumor extension into the renal vein or its segmental branches, invasion of the perirenal or renal sinus fat, or involvement of the pelvicalyceal system but not beyond Gerota's fascia, irrespective of tumor size. T3a RCC exhibited a higher recurrence risk compared to organ-confined T1-T2 lesions after surgery (6). Contemporary studies regarding the oncological outcomes of PN compared to RN for non-metastatic pT3a RCC remain inconsistent (7-11), and most of these studies are limited by a lack of adjustment for invasion patterns, as well as insufficiency of functional outcomes. While emerging evidence suggested that robot-assisted PN (RAPN) was feasible and safe for selected cT3a masses, considerable debate persisted regarding the expansion of PN indications to patients with cT3a RCC across all invasion patterns, particularly in the absence of a compelling indication for nephron preservation (12).

With the growing experience in the minimally invasive (MIS) surgery(13, 14), robust comparative data evidence specifically evaluating MIS-PN versus MIS-RN in pT3a RCC remains scarce. Thus, we conducted a multicenter, matched-cohort study to compare mild-term oncological and functional outcomes between MIS-PN and MIS-RN in patients with non-metastatic pT3a RCC.

## MATERIALS AND METHODS

### Patient population

With approval from the Institutional Review Board (approval number: B2025-341), a retrospective review was conducted on patients who underwent MIS (robot-assisted or laparoscopic) surgery for pT3a RCC between June 2016 and November 2023 at five tertiary centers in China. All patients received a comprehensive preoperative evaluation, including physical examination, laboratory testing, abdominal cross-sectional imaging (CT or MRI), and chest imaging (X-ray or CT). All procedures were performed by urological oncologists, and the choice of surgical approach (MIS-PN or MIS-RN) was determined based on patient characteristics and surgeon preference. Exclusion criteria included: reoperation for recurrent RCC; radiologic evidence of distant metastasis (cM1), lymph node metastasis (cN1), or pathologically confirmed metastasis (pN1/pM1); receipt of neoadjuvant therapy.

Patients underwent their first follow-up assessment at 3 months postoperatively, followed by surveillance every 6 to 12 months. Each follow-up included physical examination, laboratory testing, abdominal cross-sectional imaging (CT or MRI), and chest imaging (X-ray or CT). The detection of a new lesion on imaging was considered a recurrence, and the diagnosis was confirmed histologically. Lesions occurring adjacent to the resection site were classified as local recurrence, while metachronous lesions in the ipsilateral kidney (away from the resection bed) or in the contralateral kidney were not regarded as recurrences. Lesions identified in distant organs were classified as metastases.

### Data collection

Recorded preoperative features included demographic variables (age, sex, body mass index [BMI]), comorbidities (hypertension [HTN], diabetes mellitus [DM] and chronic kidney disease [CKD]), radiologic tumor characteristics (clinical tumor size and the R.E.N.A.L. score (15)). Perioperative details recorded included operative time (OT), surgical approach, intraoperative transfusion, estimated blood loss (EBL), length of stay (LOS), postoperative complications and the status and regimen of adjuvant therapy. Histopathological features included pathological tumor size, histological subtype (clear cell RCC [ccRCC] or non-ccRCC), grade (according to WHO/ISUP (16), including G1–G4 and Gx), invasion pattern (as defined by 8th AJCC/TNM criteria (5), including perinephric fat invasion[PFI], sinus fat invasion[SFI], pelvicalyceal system invasion[PSI], and renal vein invasion[RVI]), surgical margin(SM) status, and the presence of sarcomatoid differentiation (SMD), necrosis, and lymphovascular invasion(LVI). Positive SM was defined as tumor extension to the inked surface of the resected specimen. Aggressive pathological features included positive SM, grade ≥ 3, necrosis, LVI and SMD.

Renal function was assessed using serum creatinine-based estimated glomerular filtration rate (eGFR), calculated via the Chronic Kidney Disease Epidemiology Collaboration (CKD-EPI 2021) equation (17). eGFR was recorded at three time points: pre-operation (pre-GFR, within 1 month before surgery), new baseline (NB-GFR, 1–12 months postoperatively (18)), and latest follow-up (latest-GFR, >12 months postoperatively). Survival status was recorded at last follow-up.

### Statistical Analysis

Patients were stratified by the surgical approach (MIS-PN vs. MIS-RN). To minimize selection bias, two statistical adjustment methods were applied: 1) 1:2 nearest-neighbor propensity score matching (PSM) with a caliper of 0.1 (19), and 2) stabilized inverse probability of weighting (IPW) with weight truncation at the 1st and 99th percentiles (20), which was performed as a sensitivity analysis. Propensity scores were generated based on age, comorbidities (HTN/DM/CKD), clinical tumor size, R.E.N.A.L. score, and pre-GFR.

The primary outcome was recurrence-free survival (RFS). Secondary outcomes included CKD-S (pre-GFR>60 mL/min/ 1.73 m^2^ but NB-GFR <60 mL/min/ 1.73 m^2^), CKD-S3b (pre-GFR>60 mL/min/ 1.73 m^2^ but NB-GFR <45 mL/min/ 1.73 m^2^) and CKD-S at the latest follow-up (pre-GFR > 60mL/min/1.73m2 but latest-GFR < 60mL/min/1.73 m^2^). Additional renal functional endpoints included ΔGFR (NB-GFR–pre-GFR), eGFR preservation rate% (NB-GFR/pre-GFR×100%). Multivariable analyses (MVA) were performed to identify predictors of oncological and functional outcomes:1) Cox regression model was used to assess association between surgical approach (MIS-RN vs MIS-PN) and RFS, with candidate variables selected a priori (21). All results were summarized as hazard ratios (HRs) with 95% confidence intervals (CIs), and 2) Logistic regression models were applied for CKD-S, CKD-S3b, and CKD-S at the latest follow-up, estimating odds ratios (OR) with 95% CIs for variables of interest, including age, sex, BMI, tumor complexity, comorbidities, surgical approach, pathological tumor size, and pre-GFR.

Descriptive statistics were performed for patient characteristics as frequencies for categorical variables, means with standard deviations (SDs) or medians with interquartile ranges (IQRs) for continuous variables. Group comparisons between MIS-PN and MIS-RN were made using chi-square or Fisher's exact tests for categorical variables, and Mann–Whitney U tests or independent t-tests for continuous variables. Kaplan–Meier survival analyses were performed, and comparisons were assessed using the log-rank test. All p values were two tailed, with p less than 0.05 considered statistically significant. All statistical analyses were performed using R software version 4.2.2 (R Foundation for Statistical Computing, Vienna, Austria) and GraphPad-Prism version-8.3.0 (GraphPad Software, San Diego, CA, USA).

## RESULTS

### Patient characteristics

According to the inclusion and exclusion criteria, a total of 303 patients with pT3a RCC were enrolled, comprising 113 (37.3%) patients who underwent MIS-PN and 190 (62.7%) who received MIS-RN. Clinicopathological characteristics are detailed in Table-S1. The median age of the overall cohort was 58.0 years, and 73.8% of the patients were male. Compared to the MIS-PN group, patients in the MIS-RN group were slightly older (58.5 vs. 56.0 years, p=0.02), and presented with larger (6.3 vs. 3.9 cm, p <0.01), higher R.E.N.A.L. score (10.0 vs. 7.0, p <0.01) tumors, as well as a significantly higher proportion of cT3a lesions (83.2% vs. 38.9%, p<0.01). Additionally, the MIS-RN group exhibited a higher incidence of LVI compared to the MIS-PN group (21.2% vs. 9.7%, p=0.01). After PSM (66 MIS-PN/54 MIS-RN), no significant differences were observed between two groups in terms of age, comorbidities, R.E.N.A.L. score, clinical tumor size and pre-GFR. Patients undergoing MIS-PN were more likely to present with PFI, whereas those in the MIS-RN group more commonly exhibited SFI, RVI and multifocal invasion. Table-1 demonstrates clinicopathological features after PSM.

**Table 1:** Patients clinicopathological features stratified by surgical type after PSM.

Variable		PSM		
		MIS-PN(n=66)	MIS-RN(n=54)	p-value
Median age, years (IQR)		57.0 (48.3, 63.0)	58.5 (49.0, 66.0)	0.29
Male, n (%)		50 (75.8)	41 (75.9)	1.00
Median BMI, kg/m^2^, (IQR)		25.3 (23.3, 26.8)	24.28 (23.1, 26.1)	0.29
Comorbidity, n (%)		31 (47.0)	30 (55.6)	0.45
Median clinical tumor size, cm (IQR)		4.7 (4.0, 5.7)	5.1 (4.0, 6.0)	0.23
**Clinical T stage, n (%)**				**0.09**
	cT1-2	28 (42.4)	14 (25.9)	
	cT3a	38 (57.6)	40 (74.1)	
Median R.E.N.A.L. score (IQR)		8.0 (7.0, 9.0)	9.0 (7.0, 10.0)	0.49
**Tumor complexity, n (%)**				**0.49**
	Low	6 (9.1)	7 (13.0)	
	Moderate	46 (69.7)	32 (59.3)	
	High	14 (21.2)	15 (27.8)	
Preoperative eGFR, mL/min/1.73m^2^(IQR)		96.0(83.9, 103.9)	96.4(79.2, 105.0)	0.93
Median pathological tumor size, cm (IQR)		4.3 (3.3, 5.5)	5.0 (3.5, 5.7)	0.26
**Histology, n (%)**				**0.43**
	ccRCC	50 (75.8)	45 (83.3)	
	Non-ccRCC	16 (24.2)	9 (16.7)	
SMD, n (%)		3 (4.5)	3 (5.6)	1.00
Necrosis, n (%)		17 (25.8)	10 (18.5)	0.47
LVI, n (%)		7 (10.6)	11 (20.4)	0.22
**Grade, n (%)**				**0.73**
	G1-2	34 (51.5)	27 (50.0)	
	G3-4	22 (33.3)	21 (38.9)	
	Gx*	10 (15.2)	6 (11.1)	
**Invasion pattern for pT3a, n (%)**				**<0.01**
	PFI	49 (74.2)	5 (9.3)	
	SFI	7 (10.6)	28 (51.9)	
	PSI	3(4.5)	3(5.6)	
	RVI	4(6.1)	4(7.4)	
Multifocal invasion		3 (4.5)	14 (25.9)	
Adjuvant therapy, n (%)		13(19.7)	6(11.1)	0.30

PSM= propensity score matching; MIS-RN= minimally invasive radical nephrectomy; MIS-PN= minimally invasive partial nephrectomy; BMI=body mass index; eGFR=estimated glomerular filtration rate; RCC=renal cell carcinoma; OT=operation time, EBL=estimated blood loss, LOS= length of stay, ccRCC=clear cell RCC; SMD=sarcomatoid differentiation; LVI= lymphovascular invasion; SM=surgical margin; PFI=perinephric fat invasion; SFI =sinus fat invasion; PSI=pelvicalyceal system invasion; RVI=renal vein invasion; R.E.N.A.L.=[R]adius, tumor size as maximal diameter; [E]xophytic/endophytic properties of tumor; [N]earness of tumor deepest portion to collecting system or sinus; [A]nterior/Posterior [p] descriptor; and [L]ocation relative to polar line.

* Gx indicates missing or unclassified data on nuclear grade

### Survival analysis

**Figure 1:**
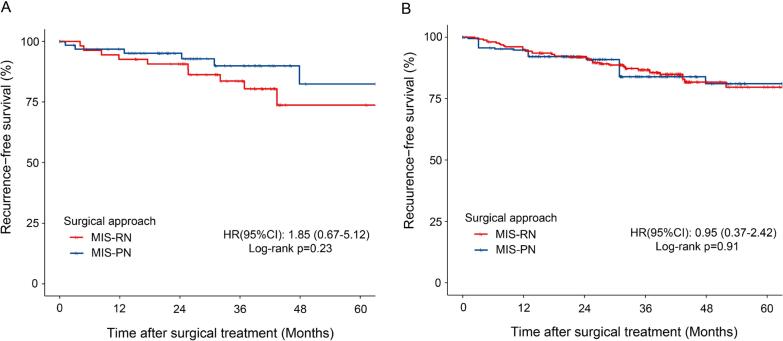
Kaplan-Meier survival analyses comparing recurrence-free survival between MIS-RN and MIS-PN.

The median follow-up was 39.0 (IQR: 26.8-52.9) months, with 34.9 (IQR: 21.6-52.9) months in MIS-PN group and 40.3 (IQR: 31.1-52.3) months in MIS-RN group. During this mild term oncologic follow up period, recurrence was observed in 42 patients (13.9%), including 9 in the MIS-PN group and 33 in the MIS-RN group. Among these, 40 cases were classified as distant metastases (7 MIS-PN/33 MIS-RN), while 2 cases were identified as local recurrence, both in the MIS-PN group. After PSM and IPW, no significant difference in RFS was observed between MIS-PN and MIS-RN group (Figures 1A and B). However, the 3-year RFS rate was significantly lower in the MIS-RN group compared to the MIS-PN group before matching (83.7% vs. 92.7%, p = 0.047) (Figure-S1).

(A) recurrence-free survival after PSM; (B) recurrence-free survival after IPW.

MIS-RN = minimally invasive radical nephrectomy; MIS-PN = minimally invasive partial nephrectomy; PSM = propensity score matching; IPW = inverse probability of weighting

MVA Cox regression analyses are depicted in Table-2. MVA analyses revealed that surgical approach (MIS-RN vs. MIS-PN) was not significantly associated with RFS in PSM cohort (HR: 1.00, p = 1.00). Larger pathological tumor size (HR: 1.42, p= 0.02) and the presence of ≥2 aggressive pathological features (HR: 4.25, p= 0.01) were independently associated with worse RFS, and the IPW analysis yielded similar results. Considering the residual heterogeneity in invasion patterns, a sensitivity analysis was conducted in the subgroup of patients with fat invasion (99 MIS -PN/105 MIS-RN). In this subgroup, surgical approach remained non-significant as a prognostic factor for RFS after both PSM and IPW.

**Table 2:** Multivariable cox regression of variables associated to recurrence-free survival in whole and fat invasion cohort after PSM and IPW.

Variable	Ref	PSM		IPW	
		HR (95%CI)	p-value	HR (95%CI)	p-value
**The whole cohort**					
Age		1.01(0.95-1.07)	0.84	1.01(0.98-1.05)	0.45
Comorbidities	No	1.93(0.45-8.24)	0.37	2.31(0.97-5.53))	0.06
High tumor complexity	Low/moderate	0.98(0.26-3.76)	0.98	0.96(0.46-1.98)	0.91
MIS-RN	MIS-PN	1.00(0.24-4.15)	1.00	0.49(0.12-2.01)	0.32
ccRCC	Non-ccRCC	0.42(0.11-1.63)	0.21	0.72(0.31-1.65)	0.44
Pathological tumor size		1.42(1.05-1.91)	0.02	1.18(0.99-1.40)	0.07
≥2 aggressive pathological features*	Negative	4.25(1.35-13.42)	0.01	2.94(1.41-6.10)	<0.01
Invasion pattern for pT3a					
SFI/PSI/RVI	PFI	2.87(0.61-13.47)	0.18	2.21(0.59-8.33)	0.24
Multifocal invasion		0.29(0.03-2.68)	0.28	1.46(0.42-5.08)	0.56
Adjuvant therapy	No	2.97(0.64-13.78)	0.16	1.40(0.47-4.16)	0.55
**Fat invasion subgroup**		HR (95%CI)	p-value	HR (95%CI)	p-value
Age		0.99(0.92-1.07)	0.86	0.99(0.95-1.03)	0.66
Comorbidities	No	1.87(0.41-8.54)	0.42	4.07(1.24-13.4)	0.02
High tumor complexity	Low/moderate	1.09(0.20-5.89)	0.92	0.97(0.40-2.34)	0.95
MIS-RN	MIS-PN	1.11(0.16-7.42)	0.92	0.17(0.03-1.01)	0.05
ccRCC	Non-ccRCC	0.83(0.15-4.61)	0.84	0.79(0.27-2.26)	0.66
Pathological tumor size		0.84(0.54-1.29)	0.42	1.30(0.95-1.79)	0.10
≥ 2 aggressive pathological features*	Negative	1.03(0.16-6.53)	0.97	4.11(1.60-10.56)	<0.01
Invasion pattern for pT3a					
SFI	PFI	1.75(0.29-10.39)	0.54	5.32(1.14-24.89)	0.03
Adjuvant therapy	No	6.05(0.92-39.9)	0.06	1.52(0.36-6.49)	0.57

MIS-RN= minimally invasive radical nephrectomy; MIS-PN= minimally invasive partial nephrectomy; ccRCC= clear cell renal cell carcinoma; SFI= sinus fat invasion; PSI= pelvicalyceal system invasion; RVI= renal venous invasion

* Aggressive pathological features: positive surgical margin, nuclear grade ≥ 3, necrosis, lymphovascular invasion or sarcomatoid differentiation

### Functional analysis

Renal functional outcomes are detailed in Table-S2. Among patients with available NB-GFR (83 MIS-PN/139 MIS-RN). no significant difference was observed in pre-GFR between MIS-PN and MIS-RN group (95.98 vs. 92.06 mL/min/1.73 m², p =0.21). However, MIS-RN was associated with a significantly greater decline in renal function, reflected by a larger ΔGFR (25.85 vs 2.59 mL/min/1.73 m², p < 0.01), a lower NB-GFR (66.42 vs.95.92 mL/min/1.73 m², p<0.01), and a reduced eGFR preservation rate (71.47% vs. 97.53%, p < 0.01). The incidences of CKD-S and CKD-S3b were both significantly higher in the MIS-RN group (both p<0.01). Among patients with available latest-GFR (83 MIS-PN/139 MIS-RN), those who underwent MIS-RN demonstrated significantly lower latest-GFR compared with MIS-PN (65.91 vs. 85.97 mL/min/1.73 m², p < 0.01) and a higher proportion of CKD-S at the latest follow-up (33.3% vs. 14.3%, p = 0.01).

**Figure 2:**
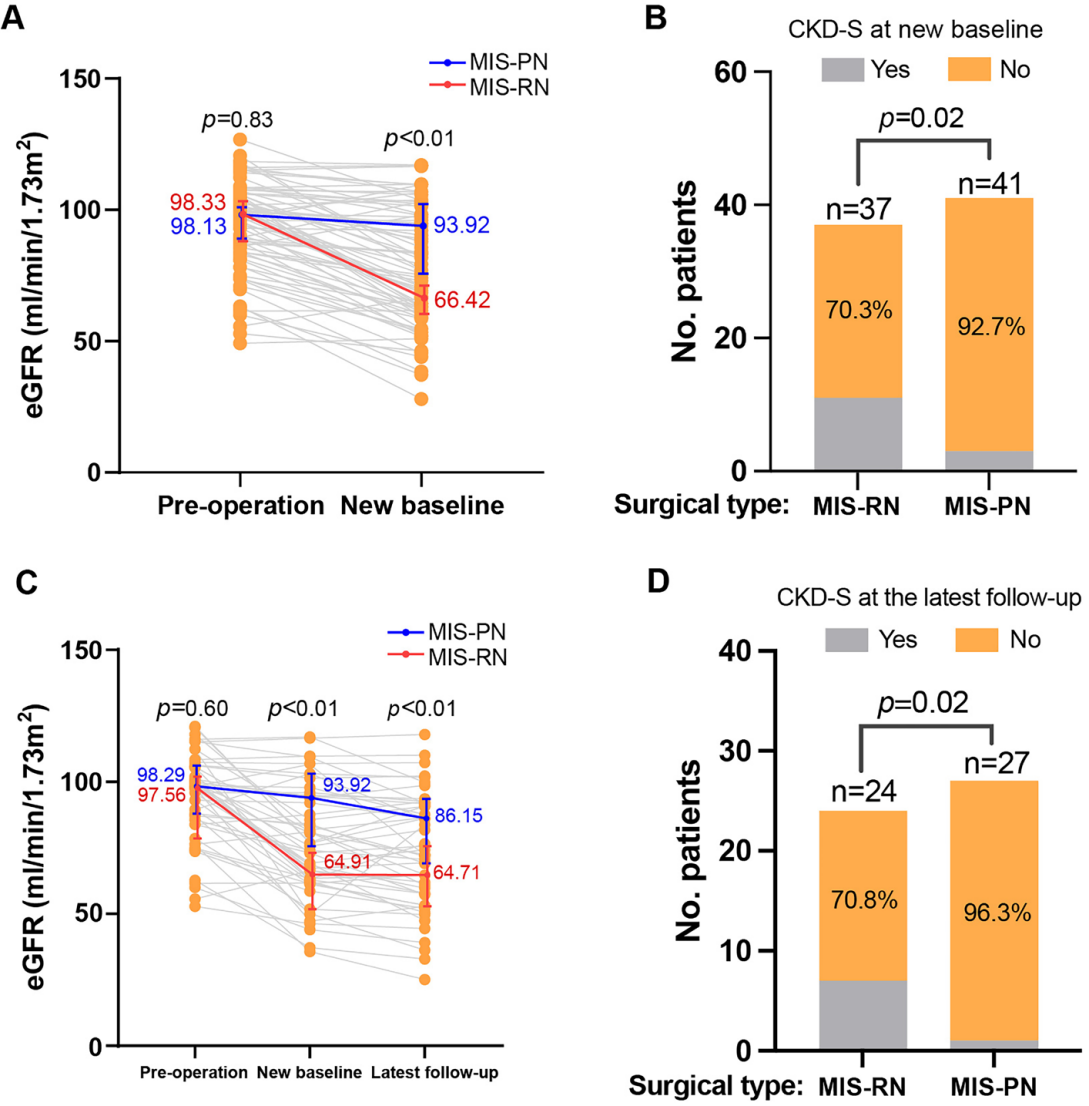
Comparison of renal functional outcomes between MIS-PN and MIS-RN groups after PSM.

Among patients with available NB-GFR after PSM (37 MIS-RN/ 41 MIS-PN), the MIS-PN group demonstrated significantly better postoperative renal functional outcomes. Specifically, MIS-PN was associated with a higher NB-GFR (93.92 vs. 66.42 mL/min/1.73 m², p < 0.01) (Figure 2A), a smaller ΔGFR (4.74 vs. 27.59 mL/min/1.73 m², p < 0.01), a lower incidence of CKD-S (7.3% vs. 29.7%, p = 0.02) (Figure 2B), and a greater eGFR preservation rate (94.92% vs. 69.42%, p < 0.01). Among patients with available latest-GFR after PSM (24 MIS-RN/27 MIS-PN), the MIS-PN group had a significantly higher NB-GFR (93.92 vs. 64.91 mL/min/1.73 m², p<0.01), latest-GFR (86.15 vs. 64.71 mL/min/1.73 m², p < 0.01), and a lower incidence of CKD-S (3.7% vs. 29.2%, p= 0.02) at the latest follow-up (Figures 2C and D) compared to the MIS-RN group.

(A) Changes in eGFR from preoperative values to the new baseline. Data are presented as the individuals and the median with interquartile range. (B) Proportion of patients with CKD-S in each group. CKD-S is defined as CKD primarily due to surgical removal of nephrons (preoperative eGFR>60 mL/min/1.73 m^2^ but new baseline eGFR <60 mL/min/ 1.73 m^2^). (C) Changes in eGFR from preoperative values to the new baseline and the latest follow-up. Data are presented as the individuals and the median with interquartile range. (D) Proportion of patients with CKD-S at the latest follow-up in each group. CKD-S at the latest follow-up is defined as preoperative GFR>60 mL/min/1.73 m2 but the latest follow-up GFR <60 mL/min/ 1.73 m^2^.

MIS-RN= minimally invasive radical nephrectomy; MIS-PN= minimally invasive partial nephrectomy; PSM=propensity score matching; eGFR= estimated glomerular filtration rate

MVA logistic regression analyses were performed to identify independent predictors of CKD-S and CKD-S3b at new baseline, as well as CKD-S at the latest follow-up (Table-3). In the PSM cohort, MIS-RN remained an independent predictor of CKD-S both at new baseline and the latest follow-up, and the IPW analysis yielded similar results. In addition, the IPW analysis showed that MIS-RN (OR: 18.29, p< 0.01), high tumor complexity (OR: 6.91, p< 0.01), smaller pathological tumor size (OR: 0.69, p= 0.01), lower pre-GFR (OR: 0.92, p< 0.01) were significant predictors of CKD-S3b at new baseline (OR: 18.29, p< 0.01) (Table-3). Clinicopathological characteristics of patients with renal function follow-up after PSM are summarized in Table-S3.

**Table 3:** Multivariable logistic regression of variables associated with CKD-S3b,CKD-S at new baseline, and CKD-S at latest follow-up after PSM and IPW.

Subgroup and variable	Ref	PSM		IPW	
		OR (95%CI)	p-value	OR (95%CI)	p-value
**CKD-S3b at new baseline**					
Age		n/a *	-	1.05(0.99-1.12)	0.12
Male	Female	n/a *	-	1.45(0.42-4.96)	0.56
BMI		n/a *	-	1.10(0.91-1.33)	0.34
High tumor complexity	Low/moderate	n/a *	-	6.91(2.10-22.79)	<0.01
Comorbidity	No	n/a *	-	2.68(0.82-8.75)	0.11
Pathological tumor size		n/a *	-	0.69(0.51-0.92)	0.01
Preoperative eGFR		n/a *	-	0.92(0.89-0.96)	<0.01
MIS-RN	MIS-PN	n/a *	-	18.29(2.53-132.01)	<0.01
**CKD-S at new baseline**					
Age		1.14(1.00-1.30)	0.04	1.05(1.01-1.09)	0.02
Male	Female	0.53(0.05-6.02)	0.61	1.27(0.54-2.97)	0.58
BMI		1.30(0.96-1.77)	0.09	1.06(0.95-1.20)	0.30
High tumor complexity	Low/moderate	2.43(0.24-24.36)	0.45	1.17(0.51-2.67)	0.71
Comorbidity	No	1.31(0.19-9.23)	0.79	1.10(0.50-2.39)	0.81
Pathological tumor size		0.63(0.34-1.14)	0.13	0.90(0.75-1.09)	0.29
Preoperative eGFR		0.94(0.90-0.99)	0.03	0.96(0.94-0.98)	<0.01
MIS-RN	MIS-PN	7.96(1.29-49.06)	0.03	5.67(2.07-15.48)	<0.01
**CKD-S at the latest follow-up**					
Age		1.08(0.96-1.21)	0.19	1.02(0.98-1.07)	0.35
Male	Female	4.80(0.59-39.13)	0.14	2.09(0.69-6.35)	0.20
BMI		1.08(0.75-1.57)	0.67	0.97(0.84-1.11)	0.64
High tumor complexity	Low/moderate	2.00(0.21-19.15)	0.55	0.51(0.18-1.42)	0.20
Comorbidity	No	0.63(0.11-3.70)	0.61	1.13(0.45-2.86)	0.80
Pathological tumor size		0.39(0.18-0.87)	0.02	1.06(0.86-1.32)	0.57
Preoperative eGFR		0.98(0.92-1.05)	0.57	0.95(0.92-0.97)	<0.01
MIS-RN	MIS-PN	7.98(1.29-51.07)	0.03	6.89(2.06-23.05)	<0.01

MIS-RN= minimally invasive radical nephrectomy; MIS-PN= minimally invasive partial nephrectomy; BMI=body mass index; eGFR=estimated glomerular filtration rate

n/a: not calculated due to low event numbers.

## DISCUSSION

The selection of treatment strategies for non-metastatic RCC is dependent on disease staging, with options ranging from RN, PN, ablation, and active surveillance(1, 2, 22, 23). With increasing experience and advancements in surgical techniques, treatment paradigms have gradually shifted. MIS-PN has been increasingly applied to larger, more complex, and even more aggressive renal tumors, with their feasibility in such cases well demonstrated (7, 24–26). However, given the morphological and biological heterogeneity of T3a RCC, MIS-PN remains challenging and controversial. We employed a robust matching design to compare oncological and renal functional outcomes of pT3aN0M0 RCC patients treated with MIS-PN versus MIS-RN. Our findings demonstrated that MIS-PN achieved non-inferior mild term oncological efficacy compared to MIS-RN in selected patients, with superior renal function preservation. Furthermore, our subgroup analysis for fat invasion also demonstrated equivalent oncological outcomes of MIS-PN vs. MIS-RN.

Existing evidence shows inconsistent results across comparative studies when all T3a invasion patterns are analyzed in aggregate. Capitanio et al. (11) compared 309 cT1aN0M0 patients pathologically upstaged to pT3a (71 PN / 238 RN) and found a significantly higher rate of PFI in the PN group (82.1% vs. 43.6%, p<0.001). After matching, MVA indicated that PN was not associated with an increased risk of metastatic progression (HR: 0.5, p=0.3) or cancer-specific mortality (CSM) (HR 0.6, p=0.4). Similarly, in a retrospective matched analysis of 140 pT3aN0M0 RCC patients (70 RAPN/70 RN), Andrade et al.(21) reported comparable 3-year cancer-specific survival (94% vs. 95%, p=0.78), OS (90% vs. 84%, p=0.42), and RFS (95% vs. 100%, p=0.06), with PFI as the predominant invasion pattern in the RAPN group (50% vs. 22.9%, p < 0.001). Pecoraro et al. (11) compared PN and RN in non-metastatic pT3a RCC using the Surveillance, Epidemiology, and End Results database and found that RN was associated with increased CSM (HR 2.10, p<0.001) in the propensity score-adjusted multivariable competing-risks regression model. Notably, PFI was more frequent in the PN group (70.3% vs. 36.4%) among patients with clearly defined pT3a invasion patterns (734 PN/ 3442 RN) before matching. However, a study by Shah et al. (8) reported contrasting findings, where patients undergoing PN for upstaged pT3a RCC from cT1 had significantly worse RFS compared to those receiving RN (HR = 5.39, p = 0.001). This discrepancy may reflect that more than 20% of cases in the PN group exhibited more aggressive T3a phenotypes (SFI or/and RVI). T3a RCC is highly heterogeneous, and evidence indicates that fat invasion carries a better prognosis than cases with RVI or multifocal invasion (27–30). Meta-analysis has shown that SFI is linked to higher CSM than PFI (31), likely due to the dense vascular and lymphatic networks in the renal sinus which increase the risk of tumor dissemination (32). In the present study, the invasion pattern in the MIS-PN cohort was predominantly characterized by PFI, which may partly explain why MIS-PN demonstrated favorable oncologic performance.

Our findings, showing that ≥2 aggressive pathological features independently predicted poorer RFS in both the PSM and IPW cohorts, closely align with prior studies(10,21). Notably, adjuvant therapy failed to improve RFS, contrasting with the KEYNOTE-564 trial (33). This discrepancy may reflect selection bias and the limitations of sample size and follow-up. Although Garofano et al. (34) suggested a potential benefit of adjuvant therapy in patients with positive SM, low positive SM rate in our study may also have reduced the ability to detect such an effect.

Compared with studies focusing on survival outcomes, research on renal functional outcomes following PN vs. RN in pT3a RCC patients remains limited. Andrade et al. (21) reported that renal function preservation rate was higher in the RAPN group than in the RN group at 3-6 months postoperatively (86.0% vs. 70.0%, p < 0.001). Similarly, Patel et al. (9) found that PN was associated with a smaller ΔGFR (6.1 vs. 19.4 mL/min/1.73 m², p < 0.001) and a lower incidence of new-onset CKD stage 3 at last follow-up (9.5% vs. 21%, p = 0.008) in upstaged T3a RCC patients who underwent PN. Our study also confirmed the advantages of MIS-PN in the renal function preservation, consistent with prior findings comparing PN and RN for T1/T2 tumors (35). Moreover, we identified RN as an independent risk factor for both CKD-S and CKD-S3b. The RENSAFE score developed by Saitta et al. (36) identified RN (HR 2.2, p <0.01) as an independent risk factor for developing de novo CKD ≥ 3b. In a subsequent analysis, Saitta et al. (10) further demonstrated that RN (HR 1.67, p = 0.025) was independently associated with a higher risk of CKD-S3b in pT3aN0M0 RCC patients. A key distinction lies in our primary reliance on the NB-GFR for assessing renal functional outcomes. Previous studies have shown that all-cause mortality is slightly higher in patients with CKD-S compared to those without CKD (HR 1.19, p = 0.030) and established that patients with NB-GFR < 45 mL/min/1.73 m² exhibit higher rates of progressive renal function decline and all-cause mortality (37, 38). Collectively, these findings encourage the consideration of PN for pT3aN0M0 patients when effective tumor control can be achieved.

While our findings corroborate the feasibility of MIS-PN for patients with pT3aN0M0 RCC, the surgical management of cT3a RCC requires careful consideration of tumor characteristics (size, location, and invasion patterns) and renal function. In our study, pathological upstaging occurred more frequently in the MIS-PN group versus the MIS-RN group (61.1% vs. 16.8, p<0.001). This discrepancy can be attributed to the substantially high proportion of PFI in the MIS-PN group, given that PFI is typically undetectable via preoperative imaging, with a reported diagnostic sensitivity of only 32% (39). Conversely, tumors in the MIS-RN cohort tended to be larger, more complex and biologically more aggressive. These characteristics accounted for the inferior RFS observed in the MIS-RN group before matching. Although Saitta et al. (10) revealed comparable survival outcomes among patients with upstaged and non-upstaged pT3aN0M0 RCC (HR 0.86, p=0.69), their study lacked detailed data regarding specific T3a invasion patterns, precluding stratified efficacy analysis. Yim et al. (40) reported that RAPN for cT3a tumors achieved a 5-year RFS of 82%, with 66.2% of patients considered to have fat invasion. However, their study did not include a comparison with RN. Our study confirmed the therapeutic equivalence of MIS-PN and MIS-RN (HR: 1.11, p=0.92) in the fat invasion subgroup. That said, given the limited sample size, further investigations in aggressive subgroups are warranted in future studies. For more aggressive T3a subtypes, although Morgan et al. (41) reported favorable outcomes for 45 pT3N0M0 RCC patients with venous tumor thrombus treated with RAPN, with 2-year local recurrence-free and metastasis-free survival rates of 95.4% and 95.3%, respectively, the follow-up period was relatively short and no comparison with RN was conducted. The limitations of existing evidence further underscore the necessity of implementing tailored treatment strategies. In the absence of high-quality evidence-based data, RN may be preferred for cT3a RCC with suspected RVI or multifocal invasion if the contralateral kidney functions normally and the NB-GFR is expected to exceed 45 mL/min/1.73m². PN should only be considered in carefully selected patients with suspected fat invasion, particularly those with PFI. In addition, surgeon expertise should be fully considered in clinical decision-making.

This study has several limitations due to its retrospective nature. The choice of surgical approach was influenced by tumor characteristics, introducing inherent selection bias. Although robust matching methods were applied to mitigate this bias, the MIS-PN group was still predominantly composed of cT1–T2 RCC, whereas the MIS-RN group mainly included cT3a RCC with higher aggressiveness. While we confirmed that PN provides equivalent oncologic outcomes to RN in patients with fat invasion, the limited sample size prevents verification of such equivalence in subgroups with RVI or multifocal invasion, warranting further investigation. In addition, surgical approach selection was influenced by surgeon preference and institutional experience, and the study cohort was drawn from high-volume specialized centers, which may limit the generalizability of these findings to lower-volume centers. Furthermore, the follow-up period was relatively short, and long-term follow-up is needed to fully validate these results. Taken together, in the era of widespread adoption of minimally invasive surgery, our multicenter analysis with robust matching demonstrates that, in high-volume centers with experienced surgeons, MIS-PN for selected pT3aN0M0 RCC patients provides mild term oncologic outcomes comparable to MIS-RN, while offering superior renal function preservation.

## CONCLUSIONS

Patients with pT3aN0M0 RCC treated with MIS-PN had comparable mild term oncological outcomes compared to MIS-RN, while providing superior renal functional preservation. As such, for carefully selected cT3a RCC cases, MIS-PN may be regarded as a viable option when technically feasible and nephron preservation is indicated. Continued follow-up is warranted to further elucidate the long-term oncological safety and refine selection criteria for MIS-PN in this subset of patients.

## Data Availability

All data generated or analysed during this study are included in this published article
