# Supplementary material for: Minimally Invasive Partial versus Radical Nephrectomy for Non-metastatic pT3a Renal Cell Carcinoma: a Multicenter Matched Cohort Study
Source: Int Braz J Urol. 2026 Jan 26;52(3):e20250500. doi: 10.1590/S1677-5538.IBJU.2025.0500 (PMC13124172; doi:10.1590/S1677-5538.IBJU.2025.0500)
Supplement: APPENDIX [file 1677-6119-ibju-52-03-e20250500-suppl1.pdf]

APPENDIX

Table S1 - Clinicopathological characteristics of the overall cohort and stratified by surgical type.

Variable	Overall(n=303)	MIS-PN(n=113)	MIS-RN(n=190)	p-value
Median age, years (IQR)	58.0(49.0~65.0)	56.0 (48.0~ 63.0)	58.5 (51.3~ 66.0)	0.02
Male, n (%)	223(73.6)	88(77.9)	135(71.1)	0.24
Median BMI, kg/m2, (IQR)	24.6 (22.8~26.6)	25.2 (23.0~ 26.8)	24.5 (22.6~ 26.3)	0.16
Comorbidities, n (%)	141 (46.5)	55 (48.7)	86 (45.3)	0.65
Median clinical tumor size, cm (IQR)	5.5 (4.0~ 7.2)	3.9 (3.0~ 4.8)	6.3 (5.2~ 8.1)	<0.01
**Clinical T stage, n (%)**				<0.01
T1-2	101(33.3)	69(61.1)	32(16.8)	
T3a	202(67.7)	44(38.9)	158(83.2)	
Median R.E.N.A.L. score (IQR)	9.0(7.0~ 10.0)	7.0 (6.0~ 9.0)	10.0 (8.0~ 10.0)	<0.01
**Tumor complexity, n (%)**				<0.01
Low	51 (16.8)	43 (38.1)	8 (4.2)	
Moderate	142 (46.9)	56 (49.6)	85 (45.3)	
High	110 (36.3)	14 (12.4)	96 (50.5)	
**Laterality, n (%)**				0.94
Left	164 (54.1)	62 (54.9)	102 (53.7)	
Right	139 (45.9)	51 (45.1)	88 (46.3)	
**Surgical technique, n (%)**				<0.01
Laparoscopic	189 (62.4)	45 (39.8)	144 (75.8)	
Robot-assisted	114 (37.6)	68 (60.2)	46 (24.2)	
Mean OT, min (SD)	142.1(59.8)	149.8(70.9)	137.6(51.7)	0.09
Median EBL, mL (IQR)	50.0(20.0-100.0)	50.0(20.0-150.0)	50.0(20.0-100.0)	0.51
Mean LOS, days (SD)	5.0(1.9)	5.1(2.0)	5.0 (1.8)	0.60
Intraoperative transfusion, n (%)	19(6.3)	8(7.1)	11(5.8)	0.84
Postoperative complications, n (%)	12(4.0)	8(7.1)	4(2.1)	0.07
Median pathological tumor size, cm (IQR)	5.0 (3.5~ 6.5)	3.5 (2.8~ 4.5)	6.0 (4.6~ 7.5)	<0.01
Preoperative eGFR, mL/min/1.73m^2 (IQR)	93.1 (79.7~102.9)	96.0 (84.0~ 102.8)	91.8 (77.0~ 103.0)	0.16
**Histology, n (%)**				0.04
ccRCC	235 (77.6)	80 (70.8)	155 (81.6)	
Non-ccRCC	68 (22.4)	33 (29.2)	35 (18.4)	
SMD, n (%)	11 (3.6)	3 (2.7)	8 (4.2)	0.70
Necrosis, n (%)	79 (26.2)	22 (19.5)	57 (30.0)	0.06
LVI, n (%)	52 (17.2)	11 (9.7)	41 (21.2)	0.01
**Grade, n (%)**				0.09
G1-2	146 (48.2)	62 (54.9)	84 (44.2)	
G3-4	121 (39.9)	36 (31.9)	85 (44.7)	
Gx*	36 (11.9)	15 (13.3)	21 (11.1)	
Positive SM, n (%)	2 (0.7)	2(1.8)	-	-
**Invasion pattern for pT3a, n (%)**				<0.01
PFI	115 (38.0)	87 (77.0)	28 (14.7)	
SFI	89 (29.4)	12 (10.6)	77 (40.5)	
PSI	19(6.3)	3 (2.7)	16 (8.4)	
RVI	21(6.9)	5(4.4)	16(8.4)	
Multifocal invasion	59(19.5)	6 (5.3)	53 (27.9)	
**Adjuvant therapy, n (%)**	51 (16.8)	24 (21.2)	27 (14.2)	0.16
TKI, n (%)	10(3.3)	3(2.7)	7(3.7)	
PD-1 inhibitor, n (%)	36(11.9)	19(16.8))	17(8.9)	
TKI plus PD-1 inhibitor, n (%)	5(1.7)	2(1.8)	3(1.6)	

BMI=body mass index; eGFR=estimated glomerular filtration rate; RCC=renal cell carcinoma; OT=operation time, EBL=estimated blood loss, LOS= length of stay, ccRCC=clear cell RCC; SMD=sarcomatoid differentiation; LVI= lymphovascular invasion; SM=surgical margin; PFI=perinephric fat invasion; SFI =sinus fat invasion; PSI=pelvicalyceal system invasion; RVI=renal vein invasion; TKI= tyrosine kinase inhibitor; PD-1= programmed death protein 1; R.E.N.A.L.=[R]adius, tumor size as maximal diameter; [E]xophytic/endophytic properties of tumor; [N]earness of tumor deepest portion to collecting system or sinus; [A]nterior/Posterior [p] descriptor; and [L]ocation relative to polar line.
*Gx indicates missing or unclassified data on nuclear grade

Table S2 - Renal function outcomes within and beyond 1 year after surgery in patients undergoing MIS-PN and MIS-RN.

Subgroup and variable	MIS-PN(n=83)	MIS-RN(n=139)	p-value
**1-12 month postoperatively**			
Preoperative eGFR, mL/min/1.73m^2(IQR)	95.98(85.63,102.61)	92.06(77.34,103.28)	0.21
New baseline eGFR, mL/min/1.73m^2(IQR)	95.92(75.47,106.21)	66.42 (53.27, 77.07)	<0.01
Median △GFR, mL/min/1.73m^2(IQR)	2.59(-1.50,10.87)	25.85(17.25,33.96)	<0.01
Median eGFR preservation, %(IQR)	97.53(86.87,101.40)	71.47(63.55,81.41)	<0.01
CKD-S, n (%)	8(9.6)	48(34.5)	<0.01
CKD-S3b, n (%)	1(1.2)	18(12.9)	<0.01
Subgroup and variable	MIS-PN(n=63)	MIS-RN(n=99)	p-value
**> 1 year postoperatively**			
Latest follow-up eGFR, mL/min/1.73m^2(IQR)	85.97(71.18-98.19)	65.91(56.08,80.06)	<0.01
CKD-S, n (%)	9(14.3)	33(33.3)	0.01

eGFR: estimated glomerular filtration rate

Table S3 - Clinicopathological characteristics of patients with renal function follow-up within and beyond 1 year postoperatively, stratified by surgical type after PSM

Subgroup and variable	PSM		
**1-12 month postoperatively**	MIS-PN(n=41)	MIS-RN(n=37)	p-value
Median age, years (IQR)	57.0(49.0-63.00)	56.0(49.0-65.0)	0.92
Male, n (%)	29(70.7)	27(73.0)	1.00
Median BMI, kg/m^2, (IQR)	25.0(23.7-26.6)	24.5(22.9-26.8)	0.60
Comorbidities, n (%)	17(41.5)	16(43.2)	1.00
Median clinical tumor size, cm (IQR)	5.0(4.1-6.1)	5.6(4.0-6.3)	0.45
Median R.E.N.A.L. score (IQR)	8.0(7.0-9.0)	9.0(7.0-10.0)	0.47
**Tumor complexity, n (%)**			0.57
Low	4(9.8)	5(13.5)	
Moderate	27(65.9)	20(54.1)	
High	10(24.4)	12(32.4)	
Preoperative eGFR, mL/min/1.73m^2(IQR)	98.1(86.0-106.3)	98.3(81.0-105.4)	0.83
Median pathological tumor size, cm (IQR)	4.5(3.8-6.0)	5.0(4.0-5.7)	0.68
**>1 year postoperatively**	MIS-PN(n=27)	MIS-RN(n=24)	p-value
Median age, years (IQR)	58.0(50.5-63.0)	58.0(42.8-64.3)	0.96
Male, n (%)	18(66.7)	18(75.0)	0.73
Median BMI, kg/m^2, (IQR)	25.1(24.1-26.6)	23.9(23.0-25.9)	0.14
Comorbidities, n (%)	11(40.7)	11(45.8)	0.93
Median clinical tumor size, cm (IQR)	5.3(4.2-6.6)	5.6(4.9-6.5)	0.44
Median R.E.N.A.L. score (IQR)	8.0(7.0-9.0)	8.0(7.0-10.0)	0.71
**Tumor complexity, n (%)**			0.83
Low	3(11.1)	2(8.3)	
Moderate	18(66.7)	15(62.5)	
High	6(22.2)	7(29.2)	
Preoperative eGFR, mL/min/1.73m^2(IQR)	98.3(87.6-106.2)	97.6(77.9-102.9)	0.60
Median pathological tumor size, cm (IQR)	5.0(4.0-6.0)	5.0(4.0-6.0)	0.64

MIS-RN= minimally invasive radical nephrectomy; MIS-PN= minimally invasive partial nephrectomy; BMI: body mass index; eGFR: estimated glomerular filtration rate; R.E.N.A.L.=[R]adius, tumor size as maximal diameter; [E]xophytic/endophytic properties of tumor; [N]earness of tumor deepest portion to collecting system or sinus; [A]nterior/Posterior [p] descriptor; and [L]ocation relative to polar line.

Figure S1 - Kaplan-Meier survival analyses comparing recurrence-free survival between MIS-RN and MIS-PN before matching.

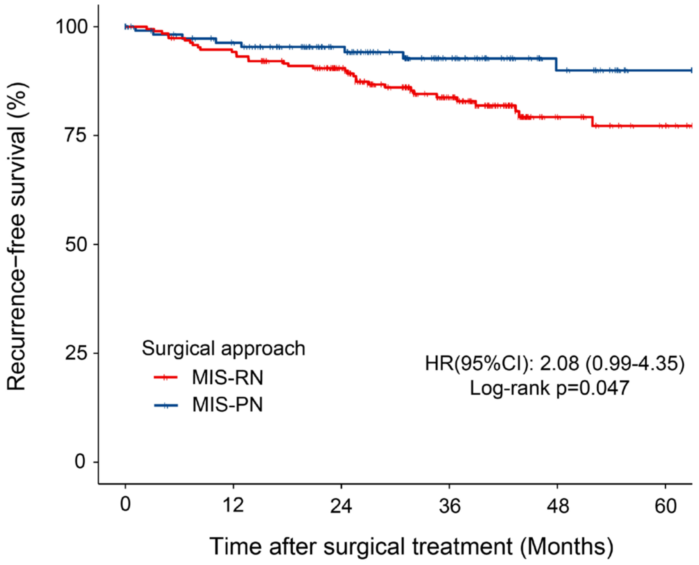

MIS-RN = minimally invasive radical nephrectomy; MIS-PN = minimally invasive partial nephrectomy
